# A Meta-Analysis of Risk Factors for Combat-Related PTSD among Military Personnel and Veterans

**DOI:** 10.1371/journal.pone.0120270

**Published:** 2015-03-20

**Authors:** Chen Xue, Yang Ge, Bihan Tang, Yuan Liu, Peng Kang, Meng Wang, Lulu Zhang

**Affiliations:** 1 Institute of Military Health Management, Second Military Medical University, Shanghai, China; 2 Faculty of Health Service, Second Military Medical University, Shanghai, China; Central Institute of Mental Health, GERMANY

## Abstract

Post-traumatic stress disorder (PTSD), a complex and chronic disorder caused by exposure to a traumatic event, is a common psychological result of current military operations. It causes substantial distress and interferes with personal and social functioning. Consequently, identifying the risk factors that make military personnel and veterans more likely to experience PTSD is of academic, clinical, and social importance. Four electronic databases (PubMed, Embase, Web of Science, and PsycINFO) were used to search for observational studies (cross-sectional, retrospective, and cohort studies) about PTSD after deployment to combat areas. The literature search, study selection, and data extraction were conducted by two of the authors independently. Thirty-two articles were included in this study. Summary estimates were obtained using random-effects models. Subgroup analyses, sensitivity analyses, and publication bias tests were performed. The prevalence of combat-related PTSD ranged from 1.09% to 34.84%. A total of 18 significant predictors of PTSD among military personnel and veterans were found. Risk factors stemming from before the trauma include female gender, ethnic minority status, low education, non-officer ranks, army service, combat specialization, high numbers of deployments, longer cumulative length of deployments, more adverse life events, prior trauma exposure, and prior psychological problems. Various aspects of the trauma period also constituted risk factors. These include increased combat exposure, discharging a weapon, witnessing someone being wounded or killed, severe trauma, and deployment-related stressors. Lastly, lack of post-deployment support during the post-trauma period also increased the risk of PTSD. The current analysis provides evidence of risk factors for combat-related PTSD in military personnel and veterans. More research is needed to determine how these variables interact and how to best protect against susceptibility to PTSD.

## Introduction

Posttraumatic stress disorder (PTSD) among military personnel and veterans has been studied for more than 30 years, PTSD may develop after an individual experiences or witnesses a traumatic event, such as combat, a natural disaster, or a violent personal assault [1]. PTSD is often been studied among military personnel in relation to combat trauma [2–5]. The effect of combat on PTSD in military personnel is a major concern among the public, military leaders, and policy makers [6], indeed, it can be a debilitating consequence of severe or life-threatening trauma [7]. Moreover, PTSD can cause substantial distress and interfere with personal and social functioning, subsequently leading to social withdrawal, anger, and aggression [8–11]. Furthermore, PTSD in military populations has a pervasive impact on military readiness and the accomplishment of military goals [12]. Studies that focus on PTSD can be divided into several categories, These categories include the basic theoretical accounts of PTSD (e.g., disease epidemiology, clinical manifestation and classification, comorbid conditions), research on pathogenic factors related to PTSD (e.g. traumatic events, family history of mental disorders, social factors); the diagnosis and evaluation of PTSD; and the treatment and prevention of PTSD. Researchers have proposed many theories to explain the development of PTSD, including biological theories, and psychological theories. Psychology theories include the psychodynamic theory as well as learning theory and cognitive theories; while cognitive theory best explains the development of PTSD [13]. The current research on the biological theory of PTSD aims to better understand the risk factor and related neurobiological mechanisms related to the illness. Neurobiological research has indicated that PTSD has distinct mechanisms that are different from the general stress response and other mental illnesses. For example, Bremner et al. noted that patients with PTSD exhibit hypothalamic-pituitary-adrenal axis (HPA axis) disorder [14, 15]. In addition, Gelpin, Bonne, et al. speculated that people with PTSD have abnormal levels of catecholamine since they demonstrate high alert symptoms such as insomnia and becoming frightened easily [16]. Moreover, Wong et al. reported that changes in immunology may be involved in the occurrence and maintain of PTSD [17].

The risk factor literature shows that not everyone who experiences a traumatic event will develop PTSD. Therefore, it has become increasingly accepted that individual vulnerability factors contribute to the development of PTSD beyond the traumatic event itself [18]. In past several decades, a number of studies have focused on combat-related PTSD and have identified individual and social risk factors, these risk factors include being younger at the time of the trauma, being female, being of a racial minority, being of a lower socioeconomic status (SES), and lack of social support [19, 20]. While all studies included routinely measured demographic factors, there is little consistency in the risk factors examined or in the measures used to assess these factors [3]. While the methods assessing trauma severity have been fairly consistent in studies of veterans, there are increasingly more studies are focusing on more specific factors that cannot be compared across studies because there are no standardized measurements of high levels of threat, the experience of atrocities, abusive violence, and neutral or malicious interpersonal atmospheres [21]. Thus, individual studies may report very different strengths of association between a given risk factor and PTSD.

Meta-analyses are able to explore variations between studies by examining how sample and study characteristics act as moderators of a given association. As such, a meta-analysis would be helpful in consolidating the abundance of information on PTSD risk factors. Kaylor et al. conducted a previous meta-analysis on the topic and focused on the general psychological impact of military service in Vietnam [22], In addition, Rubonis and Bickman, examined the positive relationship between disasters and psychopathology via meta-analysis [23]. Moreover, the meta-analysis by Weaver and Clum found that psychological distress was associated with interpersonal violence [24]. However, it must be noted that these previous meta-analyses of trauma did not systematically study the risk factors of PTSD, or include participants with specific diagnoses of PTSD [3]. In a more recent examination, Shalev summarized the results of 38 studies and found that pre-trauma vulnerability (e.g., family history of mental disorders, gender, personality traits, early traumatization, negative parenting experiences, and lower education) were associated with PTSD. Specifically, the magnitude of the stressor, preparation for the traumatic event, immediate reactions to the trauma, and post-trauma factors (e.g., emerging symptoms, social support, and other life stress) were related with PTSD [25]. Brewin et al. also studied predictors of PTSD and showed that pre-trauma risk factors have relatively weak predictive effects, while trauma intensity and post-trauma risk factors have somewhat stronger predictive effects. For instance, a lack of social support, life stress, trauma severity, childhood abuse, and other adverse childhood experiences were strong predictors of PTSD. Importantly, Brewin et al. noted that the set of studies they included in their meta-analysis was heterogeneous [3].

Drawing from Brewin et al.’s study, Ozer et al. focused on a comparison of static predictors (e.g., psychological adjustment prior to the index traumatic event, family history of psychopathology, perceived life threat during the traumatic event) to predictors that are more likely to be implicated in the psychological and neurobiological processes associated with the exposure to traumatic stress. They indicated that peri-traumatic dissociation was the strongest predictor of PTSD, followed by perceived life threat and lack of perceived support [26]. In another meta-analysis, Trickey estimated the population effect sizes of 25 potential risk factors for PTSD in children and adolescents; they indicated that subjective peri-truma factors and post-event factors played a major role in the development of PTSD [11]. However, their analysis mainly targeted studies on the risk factors of PTSD in civilian populations (children, adolescents, and adults), rather than veterans or military personnel.

Thus, more research is needed to determine the key risk factors among military personnel deployed to combat zones. A better reliable understanding of the factors that make military personnel and veterans more likely to develop PTSD can help clinicians provide the necessary treatment before difficulties become chronic [27]. Likewise, a better understanding of PTSD may make it possible to improve assessments, prevention initiatives, and interventions, thereby leading to better outcomes for military personnel and veterans exposed to potentially traumatic events [26]. In our study, we investigated and succeeded in founding the factors that may influence the development of combat-related PTSD in military personnel and veterans using meta-analytical techniques [28]. The predictive factors of PTSD included in our meta-analysis can be broadly categorized as pre-, peri-, and post-trauma factors [3, 26]. Pre-trauma factors include socio-demographic factors, military characteristics, prior traumatic experiences, and a history of psychiatric illness, whereas peri-trauma factors include combat exposure, the severity of the trauma, and acute reactions to deployment-related adverse events. Post-trauma factors include individual comorbid psychological problems, social support, and subsequent stressful life events.

## Methods

The methods and reporting procedures were in accordance with the PRISMA (Preferred Reporting Items for Systematic Reviews and Meta-Analyses) checklist. A flow diagram has been provided as supplementary material (S1 PRISMA Checklist) [29].

### Search Strategy

English-language articles published in peer-reviewed journals between 1980 (the year PTSD was first included in the Diagnostic and Statistical Manual of Mental Disorders, DSM) and April 2014 were considered for inclusion. Four psychological and medical literature databases were searched: PubMed, Embase, PsycINFO, and Web of Science. Search terms entered into the literature databases included combinations of the following: PTSD or post-traumatic stress disorder; military, or troop(s), or army, or navy, or marine corps, or air force, or armed, or defense, or peacekeeper(s), or soldier(s), or veteran(s); and, risk, predictor, prediction, or predisposition. A predictor or risk factor was operationally defined as any variable examined as a potential contributor to variability in PTSD symptom levels or diagnostic status. In addition, a manual search of references cited in all relevant original and review articles was conducted. For any full texts that were not available, we attempted to obtain information from the authors by email. This literature search yielded a preliminary database of 2,657 published articles, which were then reviewed for inclusion in the meta-analysis using various inclusion and exclusion criteria.

### Inclusion and exclusion criteria

In order to be eligible for inclusion in the meta-analysis, studies had to fulfill the following criteria: (a) investigated risk factors for PTSD in military populations after deployment to combat areas; (b) reported the odds ratios (ORs) or relative risks (RRs) and corresponding 95% confidence intervals (CIs) for risk factors in the development of PTSD; (c) included the post-deployment PTSD risk factors that we had selected; and (d) included a sample of military personnel, veterans, or both.

Articles were excluded on any of the following grounds; (a) the study measured only the acute trauma response (e.g. Acute Stress Disorder or PTSD measured before one month post-trauma) rather than PTSD, which, according to the DSM-IV-TR, can be diagnosed only after one month [30]; (b) the study used a categorical measure of PTSD—in other words, they included individuals meeting full diagnostic criteria and those with less severe post-traumatic symptoms or partial PTSD (e.g., “subsyndromal PTSD”) in the same comparison group, and contrasted them with a group exposed to the same event but without PTSD; (c) the study population consisted entirely of individuals already suffering from PTSD or from a specific comorbid psychiatric disorder (e.g., depression, Attention deficit hyperactivity disorder, substance abuse, learning disabilities) or having committed a violent offense, which would limit the generalizability of the results; (d) the study did not specifically assess DSM-defined PTSD symptoms (e.g., studies that reported only general symptoms); (e) the study contained insufficient data to calculate univariate effect sizes, and such data could not be obtained from the study author; (f) the article was a review or a qualitative study that did not present new data or only presented qualitative analyses; (g) the primary aim of the study was to investigate the efficacy of treatment; and (h) the study used a single-case design [11].

Finally, if more than one article reported data from the same sample, then the most recent and complete article was included in our meta-analysis. All eligible studies were carefully reviewed by two authors (C.X. & M.W.) to ensure decision-rule consistency, with 100% agreement.

### Data extraction and quality assessment

Data extraction was performed by two investigators (C.X. and B.T.) independently. Differences in interpretation were resolved by discussion with a third co-author (Y.G.) to produce one final coding. The following information was extracted from each eligible study: first author’s surname, year of publication, study location, trauma type, study design, method, deployment area, PTSD diagnosis, sample size, PTSD prevalence, age and gender of participants, estimated effect size (OR/RR), corresponding 95% CI, and covariates adjusted for in the statistical analysis. If a study reported several multivariable-adjusted effect estimates, we selected the estimate that adjusted for the most potential confounding variables.

Quality assessments were conducted independently by two investigators (C.X. and Y.G.) using an 11-item instrument recommended by the Agency for Healthcare Research and Quality (AHRQ) for cross-sectional studies [31] and the 9-star Newcastle-Ottawa Scale (NOS) [32] for cohort studies. Studies with eight stars or more on the AHRQ and NOS were considered to be of high quality. Where the two raters’ quality assessments differed, the original articles were re-examined along with two more co-authors (Y.L. and P. K.) until a final quality rating was agreed upon. The inter-rater reliability of the quality assessment between the two main raters (C.X. and Y.G.) was high (Cohen’s kappa statistic, k = 0.68).

### Statistical analysis

We examined risk factors for combat-related PTSD in military personnel based on the ORs and 95% CIs reported in each study. A random-effects model, which assumes that the real potential effect varies among included studies, was used to estimate the pooled RRs with 95% CIs. Heterogeneity between studies was evaluated using the χ^2^ test and I^2^ statistic, with p values lower than. 05 indicating heterogeneity and higher I^2^ values indicating greater variability among trials than would be expected by chance alone (range: 0–100%) [33]. The probability of publication bias was assessed using Egger’s regression test. If publication bias was present, we tried to evaluate the effect of the publication bias using the trim and fill method [34].

The various differences among the studies might have influenced their results. Brewin et al. examined the impact of six samples and study characteristics on the effect sizes of various risk factors (e.g., type of trauma, gender of participants, analysis by diagnostic category versus continuous score) [3]. We considered that the quality of the study, deployment locations, and nationalities of samples might contribute to variances in risk factors. First, studies of different qualities would have used different study designs, measurement methods, statistical analysis, and procedures, and therefore they would naturally have obtained differing results and different quality scores. Seventeen articles (53.1% of those included) achieved a score of seven stars or less, and thus were considered low quality. Second, different deployment years and locations suggested differences in combat exposure and trauma severity levels. Twenty-one (65.6%) articles in our meta-analysis looked at military personnel and veterans who were deployed to Afghanistan or/and Iraq after the year 2000; 11 (34.4%) articles focused on persons deployed to other areas (e.g., Rwanda, Bosnia, Vietnam, Gulf) before the year 2000. Finally, different nationalities would be associated with different socio-demographic and military characteristics—21 articles (65.6%) focused on military persons from the United States, while 11 articles focused on those from other countries, such as the United Kingdom, Australia, the Netherlands, and Israel. To examine the possible effects of these variables on the study results, we conducted three subgroup (high-quality versus low-quality study; deployment to Iraq or Afghanistan versus not deployed to Iraq or Afghanistan; and from the United States versus not from the United States) and sensitivity analyses in our meta-analysis. When risk factors were for multi-categorical variables (e.g., age, education level), we used the ORs of the highest versus the lowest category.

Stata Version 12.0 software (Stata Corp, College Station, TX) was used for all analyses and all statistical tests were two-tailed. Values of p < 0.05 were considered statistically significant.

## Results

### Characteristics of studies

**Fig 1:**
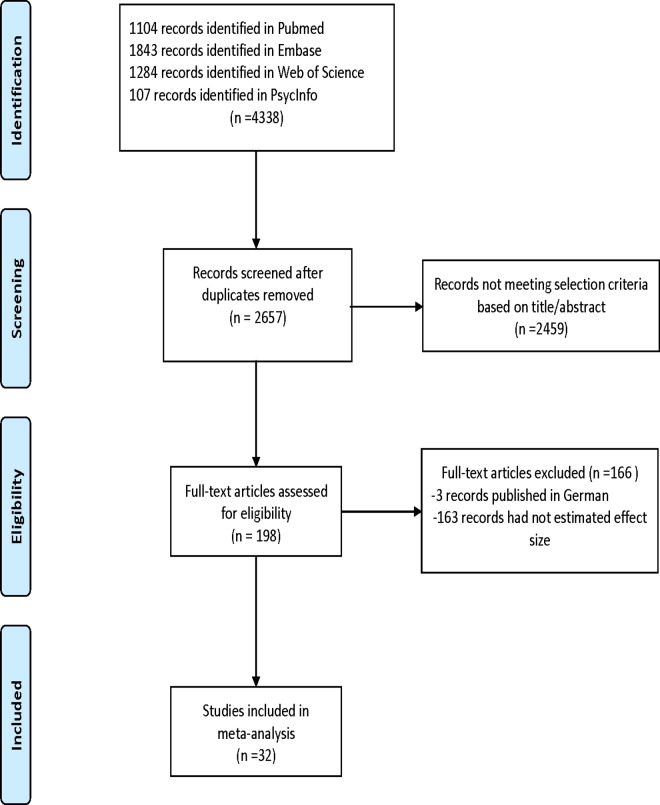
Flow of study selection.

Fig. 1 shows the complete selection process. A total of 2,657 records indexed by April 2014 were retrieved using our search strategy. We excluded 2,459 articles after reading the titles and abstracts, and retained the remaining 198 articles for further evaluation by reading the full texts. Finally, we selected 32 full-text articles about risk factors for combat-related PTSD in military populations for our meta-analysis (Fig. 1)[6, 35–65], including 25 cohort studies (4 prospectively studies, 21 retrospective studies), and 7 cross-sectional studies. Furthermore, 24 articles obtained their data through investigation (e.g., interview, questionnaire) of military personnel and veterans after they returned from their deployment areas; 4 articles prospectively studied personnel before and after deployment; and 4 articles obtained their data through a clinical data center.

Table 1 shows the general characteristics of the 32 studies retained in the analyses, including trauma type, sample size, PTSD measure, interview versus questionnaire assessment, age range, percentage of the sample that was male, and the location of the study samples. Sample sizes from individual studies ranged from 238 to 40,600. The method described above generated 27 risk factors that were explored by two or more studies and that could therefore be entered into the meta-analysis.

**Table 1:** General Characteristic of the included studies with regard to risk factors for PTSD.

Id	Author	Year	Country	Trauma type	Study design	Method	Population	Deployment area	Diagnosis	Sample size	PTSD prevalence	Male%	Age range (M,SD)	Quality
1	Macera et al.	2014	USA	Combat	Cohort	Q	Military personnel	Afghanistan or Iraq	DSM-IV	31,534	5.38%	93.99%	≥18	7
2	Harbertson et al.	2013	USA	Combat	Cross-sectional	Q	Defense Forces	Rwanda	DSM-IV	1,238	7.60%	100.00%	30.9 ± 5.6	6
3	Kline et al.	2013	USA	Combat	Cohort	I	Military personnel	Iraq	DSM-IV	922	9.65%	90.13%	31.95 ± 9.29	8
4	MacGregor et al.	2013	USA	Battle	Cohort	Q	Military personnel	Iraq	ICD-9-CM	1,777	25.15%	94.27%	≥18	7
5	Mayo et al.	2013	USA	Combat	Cohort	I	veterans	Iraq	ICD-9-CM	40,600	6.30%	100.00%	≥18	8
6	Tracie et al.	2013	USA	Combat	Cohort	I	NG Soldiers	Afghanistan or Iraq	DSM-IV-TR	238	12.60%	92.00%	33.5 ± 9.5	8
7	Van Liempt et al.	2013	Dutch	Combat	Cohort	Q	Military personnel	Afghanistan	Unspecified	453	6.6%	92.9%	28.85 ± 2.95	5
8	Goldmann et al.	2012	USA	Combat	Cohort	I	Military personnel	Afghanistan or Iraq	DSM-IV	2,616	9.60%	89.80%	≥17	7
9	Jones et al.	2012	UK	War	Cohort	Q	Military personnel	Afghanistan or Iraq	DSM-IV	8,261	4.20%	90.70%	≥18	8
10	MacGregor et al.	2012	USA	Combat	Cohort	I	Marine Corps	Iraq	ICD-9-CM	16,376	1.46%	-	17–57	8
11	Rona et al.	2012	UK	War	Cohort	Q	Military personnel	Iraq	Unspecified	6,292	3.90%	88.91%	≥18	8
12	Wells et al.	2012	USA	Combat	Cohort	Q	Military personnel	Not mention	DSM-IV-TR	11,017	4.95%	53.00%	>18	7
13	Du Preez et al.	2011	UK	War	Cohort	Q	Military personnel	Iraq	Unspecified	4,901	-	100.00%	≥18	7
14	Maguen et al.	2011	USA	War	Cross-sectional	I	veterans	Afghanistan and Iraq	DSM-IV	213,803	34.84%	87.59%	≥16	8
15	Riviere et al.	2011	USA	War	Cohort	Q	NG Soldiers	Iraq	DSM-IV-TR	5,576	>20%	67.88%	≥18	7
16	Sandweiss et al.	2011	USA	Conflicts	Cohort	Q	Military personnel	Iraq and Afghanistan	DSM-IV	22,630	8.10%	81.46%	≥18	8
17	Booth-Kewleyet al.	2010	USA	Combat	Cross-sectional	Q	Marines	Iraq and/or Afghanistan	DSM-IV	1,569	17.1%	95.00%	≥18	10
18	LeardMann et al.	2010	USA	Conflicts	Cohort	Q	Marine Corps	Iraq and Afghanistan	ICD-9-CM	8,391	2.69%	100.00%	≥17	7
19	Phillips et al.	2010	USA	Combat	Cohort	Q	Marine Corps	Afghanistan or Iraq	DSM-IV	706	10.80%	100.00%	17–31	7
20	Iversen et al.	2009	UK	War	Cross-sectional	I	Military personnel	Iraq	DSM-IV	821	4.80%	-	≥18	8
21	LeardMann et al.	2009	USA	Combat	Cohort	Q	Military personnel	Bosnia or Kosovo	DSM-IV	5,410	7.30%	84.00%	20–55	7
22	Dohrenwend	2008	USA	War	Cross-sectional	I	veterans	Vietnam	DSM-III-R	260	15.38%	100.00%	≥18	6
23	Iversen et al.	2008	UK	War	Cross-sectional	Q	Military personnel	Iraq	DSM-IV	4,762	3.72%	92.20%	≥18	8
24	Zohar et al.	2008	Israel	Combat	Cohort	I	Military Veterans	Not Mentioned	DSM-IV	2,362	-	-	18–45	6
25	Rona et al.	2007	UK	Combat	Cross-sectional	Q	Military personnel	Iraq	DSM-IV	5,547	3.66%	-	≥18	9
26	Jones et al.	2006	UK	Combat	Cohort	Q	Military personnel	Iraq	Unspecified	4,500	2.00%	92.00%	≥18	5
27	Koenen et al.	2003	USA	War	Cohort	Q	veterans	Vietnam	DSM-III-R	1,377	11.80%	100.00%	39	9
28	Barrett et al.	2002	USA	War	Cohort	I	veterans	Gulf	DSM-III-R	3,682	1.09%	89.54%	≥18	8
29	Koenen et al.	2002	USA	War	Cohort	I	veterans	Vietnam	DSM-III-R	2,708	20.80%	100.00%	36–55	8
30	O’Toole et al.	1998	Australia	Combat	Cohort	I	veterans	Vietnam	DSM-III-R	1,000	20.90%	100.00%	-	5
31	O’Toole et al.	1998	Australia	War	Cohort	I	veterans	Vietnam	DSM-III	641	30.47%	-	-	7
32	McCarren et al.	1995	USA	War	Cohort	I	veterans	Vietnam	DSM-III	2,210	11.40%	100.00%	>18	6

Note:

Abbreviations: USA = Untied States of America; Q = Questionnaire; I = Interview.

Range of the quality rating, Cohort study: 0–9; Cross-sectional study: 0–11.

### Classification of risk factors

In the 32 studies included in the analyses, the risk factors for combat-related PTSD in military personnel and veterans were as follows: (a) pre-trauma factors, including socio-demographic factors (e.g., age, gender, race, education level, marital status), military characteristics (e.g., rank, branch of service, occupation, number of deployments, length of deployments), smoking status, drinking status, low SES, prior life events, prior trauma, and prior psychological problems; (b) peri-trauma factors, including unit support, combat exposure, component, discharging a weapon, witnessing someone being wounded or killed, and trauma severity; and (c) post-trauma factors, including comorbid psychological problems, subsequent life events, and post-deployment support.

### Study outcome

**Fig 2:**
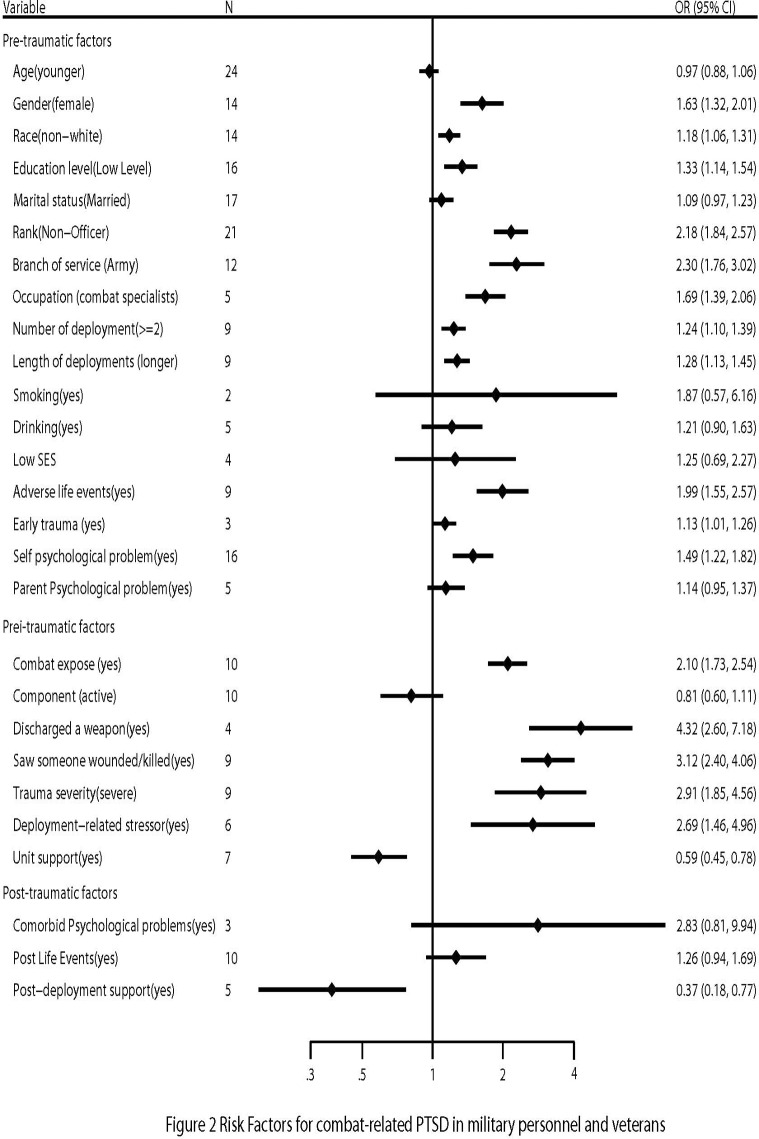
Forest plots of risk factors for combat-related PTSD in military personnel.

The prevalence of PTSD in military personnel and veterans ranged from 1.09% to 34.84%. The risk factors for combat-related PTSD are presented in Table 2 and Fig. 2. Multiple pre-trauma risk factors were associated with PTSD, including being female (OR = 1.63; 95% CI, 1.32–2.01), being non-White (OR = 1.18; 95% CI, 1.06–1.31), having low education (OR = 1.33; 95% CI, 1.14–1.54), being non-officers (OR = 2.18; 95% CI, 1.84–2.57), serving in the army (OR = 2.30; 95% CI, 1.76–3.02), more deployments (OR = 1.24; 95% CI, 1.10–1.39), a longer cumulative length of deployments (OR = 1.28; 95% CI, 1.13–1.45), experiencing adverse life events (OR = 1.99; 95% CI, 1.55–2.57), experiencing prior trauma (OR = 1.13; 95% CI, 1.01–1.26), and having prior psychological problems (OR = 1.49; 95% CI, 1.22–3.81). However, heterogeneity was found for race (I^2^ = 65.0%, p < 0.001), rank (I^2^ = 69.0%, p < 0.001), branch of service (I^2^ = 91.6%, p < 0.001), occupation (I^2^ = 70.6%, p = 0.001), number of deployments (I2 = 63.2%, p < 0.001), length of deployments (I^2^ = 71.4%, p = 0.004), experiencing adverse life events (I^2^ = 59.8%, p = 0.011), and having prior psychological problems (I^2^ = 73.9%, p < 0.001). Subgroup and sensitivity analyses indicated inconsistencies in the results for race, marital status, length of deployments, smoking status, low SES, prior trauma, and prior psychological problems (Table 2). For example, in all studies, race (being non-White) is a positive risk factor for PTSD—however, after excluding low-quality articles, race (non-White) was unrelated to the risk for PTSD. This was also true for the other aforementioned variables (e.g., marital status, smoking, prior psychological problems). Thus, the results of these studies should be interpreted with caution because of the potential bias. In addition, we found publication bias for education (Egger’s test p = 0.01), marital status (Egger’s test p = 0.013), branch of service (Egger’s test p = 0.03), length of deployment (Egger’s test p = 0.02), and prior psychological problems (Egger’s test p = 0.03). After adjusting for the publication bias, the ORs were 1.51 (95% CI, 1.28–1.79) for education, 1.09 (95% CI, 0.97–1.23) for marital status, 2.30 (95% CI, 1.76–3.02) for branch of service, 1.10 (95% CI, 0.90–1.34) for the length of deployments, and 1.49 (95% CI, 1.22–1.82) for psychological problems.

**Table 2:** Risk Factors for combat-related PTSD in military personnel and veterans.

	All studies					High quality		Deployment area(OIF/OEF)		Country(USA)	
	N	OR (95%cl)	I^2^ (P value)	Egger test	Trim and fill	N	OR (95%cl)	N	OR (95%cl)	N	OR (95%cl)
**Pre-traumatic factors**											
Age (younger)	24	0.97 (0.88–1.06)	91.6% (p < 0.001)	P = 0.256	-	15	1.00 (0.90–1.13)	11	1.03 (0.90–1.19)	18	0.96 (0.87–1.06)
Gender (female)	14	1.63 (1.32–2.01)	41.9% (p = 0.05)	p = 0.396	-	8	1.30 (1.14–1.49)	11	1.40 (1.19–1.63)	9	1.66 (1.25–2.22)
Race (non-white)	14	1.18 (1.06–1.31)	65.0% (p < 0.001)	P = 0.141	-	10	1.11 (0.99–1.23)	9	1.14 (1.02–1.28)	14	1.18 (1.06–1.31)
Education level(Low Level)	16	1.33 (1.14–1.54)	37.4% (p = 0.066)	P = 0.010	1.51 (1.28–1.79)	10	1.35 (1.08–1.72)	13	1.32 (1.32–1.32)	10	1.35 (1.18–1.56)
Marital status (Married)	17	1.09 (0.97–1.23)	81.3% (p < 0.001)	P = 0.013	1.09 (0.97–1.23)	11	1.02 (0.88–1.18)	12	1.09 (0.95–1.24)	12	1.16 (1.03–1.31)
Rank (Non-Officer)	21	2.18 (1.84–2.57)	69.0% (p < 0.001)	P = 0.454	-	13	2.08 (1.67–2.60)	16	2.15 (1.79–2.59)	13	2.05 (1.72–2.45)
Branch of service (Army)	12	2.30 (1.76–3.02)	91.6% (p < 0.001)	P = 0.030	2.30 (1.76–3.02)	9	2.59(1.93–3.46)	8	2.37 (1.73–3.24)	6	2.46 (1.72–3.51)
Occupation (combat specialists)	5	1.69 (1.39–2.06)	70.6% (p = 0.001)	P = 0.753	-	5	1.56 (1.34–1.83)	6	1.72 (1.47–2.00)	7	1.55 (1.12–1.97)
Number of deployment (≥2)	9	1.24 (1.10–1.39)	63.2% (p = 0.005)	P = 0.240	-	5	1.15 (1.13–1.18)	8	1.22 (1.09–1.37)	6	1.28 (1.13–1.45)
Length of deployments (longer)	9	1.28 (1.13–1.45)	71.4% (p = 0.004)	P = 0.020	1.10 (0.90–1.34)	4	1.02 (0.69–1.50)	8	1.21 (0.98–1.97)	8	1.22 (1.00–1.48)
Smoking (yes)	2	1.87 (0.57–6.16)	81.4% (p < 0.001)	-	-	1	3.83(1.40–10.46)	1	1.12(0.86–1.45)	1	3.83(1.40–10.46)
Drinking (yes)	5	1.21 (0.90–1.63)	59.7% (p = 0.042)	P = 0.192	-	2	1.17 (0.71–1.92)	3	1.13 (0.85–1.50)	3	1.33 (0.67–2.64)
Low SES (yes)	4	1.25 (0.69–2.27)	88.8% (p < 0.001)	P = 0.877	-	3	1.48 (0.75–2.93)	1	3.29(2.14–5.04)	3	0.97 (0.79–1.20)
Adverse life events	9	1.99 (1.55–2.57)	59.8% (p = 0.011)	P = 0.687	-	5	2.18 (1.58–3.02)	7	2.16 (1.68–2.76)	7	2.12 (1.58–2.83)
Early trauma (yes)	3	1.13 (1.01–1.26)	0% (p = 0.937)	P = 0.574	-	1	1.08 (1.08–1.34)	2	1.11 (0.95–1.31)	1	1.11 (0.94–1.31)
Psychological problem (yes)	16	1.49 (1.22–1.82)	73.9%(p < 0.001)	P = 0.030	1.49 (1.22–1.82)	3	1.01 (0.72–1.41)	10	1.34 (1.10–1.64)	10	1.36 (1.06–1.76)
Parent Psychological problem (yes)	5	1.14 (0.95–1.37)	29.1%(p = 0.228)	P = 0.743	-	0	-	1	0.88(0.60–1.29)	5	1.14 (0.95–1.37)
**Peri-trauma factors**											
Unit support (yes)	7	0.59 (0.45–0.78)	51.7% (p = 0.053)	P = 0.395	-	4	0.64 (0.47–0.87)	7	0.59 (0.45–0.78)	4	0.54 (0.38–0.76)
Combat expose (yes)	10	2.10 (1.73–2.54)	97.3% (p < 0.001)	P = 0.008	0.74 (0.55–0.93)	5	2.48 (1.40–4.41)	7	2.50 (1.83–3.41)	10	2.10 (1.73–2.54)
Component (active)	10	0.81 (0.60–1.11)	97.9% (p < 0.001)	P = 0.010	0.81 (0.60–1.11)	5	0.95 (0.64–1.41)	8	0.79 (0.57–1.11)	8	0.76 (0.54–1.06)
Discharged a weapon (yes)	4	4.32 (2.60–7.18)	90.0% (p < 0.001)	P = 0.198	-	1	2.57(1.81–3.66)	4	4.32 (2.60–7.18)	3	5.41 (3.84–7.64)
Saw someone wounded/killed (yes)	9	3.12 (2.40–4.06)	56.2% (p = 0.043)	P = 0.380	-	3	2.73 (1.73–4.31)	6	3.12 (2.40–4.06)	3	3.71 (3.35–4.11)
Trauma severity (yes)	9	2.91 (1.85–4.56)	96.8% (p < 0.001)	P = 0.480	-	4	5.11 (3.08–8.49)	8	2.92 (1.81–4.71)	9	2.91 (1.85–4.56)
Deployment-related stressor (yes)	6	2.69 (1.46–4.96)	94.3% (p < 0.001)	P = 0.057	2.69 (1.46–4.96)	4	3.09 (1.25–7.62)	5	3.11 (1.19–8.08)	4	3.08 (1.20–7.95)
**Post-trauma factors**											
Comorbid Psychological problems (yes)	3	2.83 (0.81–9.94)	92.2% (p < 0.001)	p = 0.295	-	1	1.20 (1.12–1.29)	1	2.99 (1.05–8.55)	1	1.20 (1.12–1.29)
Post Life Events (yes)	10	1.26 (0.94–1.69)	77.9% (p < 0.001)	P = 0.724	-	3	1.22 (0.69–2.15)	5	1.75 (1.45–2.12)	10	1.26 (0.94–1.69)
Post-deployment support (yes)	5	0.37 (0.18–0.77)	92.6% (p < 0.001)	P = 0.101	-	4	0.39 (0.17–0.91)	4	0.30 (0.20–0.47)	3	0.49 (0.19–1.25)

With regard to the peri-trauma risk factors affecting military personnel and veterans, those who were combat specialists were more likely to suffer from PTSD, with pooled ORs of 1.69 (95% CI, 1.39–2.05), respectively. In addition, the risk for PTSD increased with greater combat exposure (OR = 2.10; 95% CI, 1.73–2.54), the experience of discharging a weapon (OR = 4.32; 95% CI, 2.60–7.18), witnessing someone being wounded or killed (OR = 3.12; 95% CI, 2.40–4.06), severe trauma (OR = 2.91; 95% CI, 1.85–4.56), and deployment-related stressors (OR = 2.69; 95% CI, 1.46–4.96). Heterogeneity was found for unit support (I^2^ = 51.7%, p = 0.053), combat exposure (I^2^ = 97.3%, p < 0.001), component (I^2^ = 97.9%, p < 0.001), discharging a weapon (I^2^ = 90.0%, p < 0.001), witnessing someone be wounded/killed (I^2^ = 56.3%, p = 0.043), and trauma severity (I^2^ = 96.8%, p < 0.001). We found inconsistencies in the results for length of deployments in the subgroup and sensitivity analyses. We also found a publication bias for combat exposure (Egger’s test p = 0.008), and component (Egger’s test p = 0.01). After adjusting for the publication bias, the ORs were 0.74 (95% CI, 0.55–0.93) for combat exposure, and 0.81 (95% CI, 0.60–1.11) for component.

Finally, analysis of the post-trauma factors showed that comorbid psychological problems and subsequent life events were not positively related to PTSD, while post-deployment support was a protective factor (OR = 0.37, 95% CI, 0.18–0.77). However, heterogeneity was found for post-deployment support (I^2^ = 92.6%, p < 0.001), and there were inconsistencies in the results for comorbid psychological problems, subsequent life events, and post-deployment support in the subgroup and sensitivity analyses. That means military persons suffered from subsequent life events were more likely to get PTSD after they came back from Iraq or Afghanistan. This was also true for comorbid psychological problems and post-deployment support (Table 2).

## Discussion

To our knowledge, this is the first meta-analysis focusing on risk factors for combat-related PTSD in military personnel and veterans. Specifically, 27 risk factors for military PTSD were investigated across 32 observational studies (25 cohort studies, 7 cross-sectional studies) published between 1980 and April 2014. Our synthesis of the relevant articles published in English provided strong evidence of risk factors for combat-related PTSD. Approximately 11 out of 27 risk factors (41%) were investigated in ten or more studies. Although an increasing number of researchers have been studying combat-related PTSD in the past 30 years, only a limited number of variables have been routinely investigated [26]. In comparison, in a meta-analysis of risk factors for PTSD among trauma-exposed adults (based mostly on retrospective data) 11 of 14 risk factors (79%) had been considered in 10 or more studies; in that meta-analysis, psychiatric history, life stress, and other previous traumas were not strong risk factors for the development of PTSD among military service members [3]. Thus, the present study highlights the need for further investigation of some very basic potential risk factors, such as age, smoking status, and prior psychological problems.

### Pre-trauma factors

Pre-trauma factors have been reported in many studies. In this article, we focused on socio-demographic factors, military characteristics, prior psychological factors, and other prior traumatic experiences. Socio-demographic factors such as age have received attention; however, the results have been mixed [66]. Specifically, the impact of being younger at the time of trauma on the risk of developing PTSD has been difficult to predict given the contradictory effects of age on various processes underlying traumatic stress reactions [67–69]. Approximately 19 studies included in our meta-analysis examined age as a risk factor for combat-related PTSD; the results suggest that a younger age at the time of trauma is largely unrelated to PTSD. In addition, subgroup analyses revealed that there was no statistically significantly relationship between age and PTSD when only analyzing studies conducted among Americans deployed to Iraq or Afghanistan, This finding was not found in individuals deployed to other areas such as Vietnam and the Persian Gulf and for military service members from other countries. This result was somewhat different from the results published by Koenen et al. and Booth-Kewley et al. [6, 62], that indicated that a younger age was a strong risk factor for PTSD. However, in our analysis, military samples were divided into a number of diverse age brackets across 19 of the studies, therefore, we decided to code age dichotomously (younger groups vs. older groups). Thus, it is not advisable to draw conclusions about the age brackets that are vulnerable.

Over the past 20 years, the number of women serving in the military has increased, and that number is projected to continue to increase; this may increase women’s risk for developing mental health disorders [70]. Gender was also a predictor in our meta-analysis; females were more likely to experience PTSD following combat than were males. This finding supports research conducted among Army soldiers deployed to combat zones, where PTSD symptoms were found to be more common among women than in studies that compared men and women [35, 71]. A number of factors may account for these findings. However, the main reasons appear to be that women report lower military preparedness and less unit cohesion, and have higher rates of depression [72].

Moreover, it has been argued that women are more likely than are men to experience sexual assault and childhood sexual abuse; however, they are less likely to experience accidents; nonsexual assaults; and to witness death, injury, disaster, combat, and war [73]. The experience of childhood sexual abuse may exacerbate PTSD in adulthood; indeed, Breslau et al. confirmed this notion in their study using data from an epidemiologic survey. Their results were consistent with those of Brewin et al. [3, 74, 75]. Importantly, only one article (LeardMann, 2010) that met the criteria for inclusion in our meta-analysis reported relations between sexual abuse and PTSD; thus, given the low number of studies included herein, we cannot make any conclusions about the relations between childhood sexual abuse and PTSD,

Beyond the relations between abuse and PTSD, there are other reasons why females may have more PTSD. For instance, females are more sensitive to threats, less likely to use effective coping strategies, and tend to interpret trauma more negatively than males [73]. In addition, when faced with unexpected trauma, men are probably just as frightened as women are, but women are often more willing to report feeling negative emotions. This relation may also be attributed to studies that women were observed to be avoidant, on guard, easily startled, and flooded with memories and images of an assault that could not be easily dispelled [26, 76]. Females are also thought to be more sensitive to stress hormones, possibly reducing their ability to manage stressful situations [77]. Beyond hormones, it may also be that women do not benefit from some of the protective factors that benefit men; for instance, unit cohesion has been shown to benefit men more than women [78, 79].

The additional socio-demographic risk factors associated with PTSD in military personnel and veterans were race (minority ethnic groups) and education (lower education levels). Specifically, non-white military service members were more likely to suffer from PTSD than whites military service members. The reason, why minority military persons may be at risk for more negative consequences from PTSD remains under debate; however, it may be that minority individuals who serve in the military have higher levels of other pre- or post-trauma risk factors, or they may be more likely to be assigned to high combat roles. It is important to note that this variable was coded in a dichotomous fashion (white versus black/ethnic minority); therefore, important differences between minority ethnic groups may have been masked herein[3, 11]. To the point of education, different educational levels indirectly influences multiple, including economic resources, social status, social networks, and health behavior. Therefore, military service members with higher education levels may use better coping methods because they access to more resources, thereby reducing the incidence of depression. However, it is important to note that marital status did not have an effect on PTSD in this meta-analysis; this finding contradicts some previous military research. For instance, Smith et al. indicated that military persons were at a substantially higher risk for possible PTSD if they were divorced than if they were currently married or never married [6, 80].Thus, future research should explore the relationship between marital status and PTSD.

When military characteristics were examined, military rank (i.e., non-officers), branch of service (i.e., Army), occupation (i.e., combat specialists), cumulative length of deployments (i.e., longer), and number of deployments (i.e., ≥2) were important contributors to the development of combat-related PTSD. Non-officers and supply personnel were more likely to be diagnosed with PTSD; this may be because of their increased combat exposure [39]. Other studies have also show that PTSD is higher among enlisted personnel than among officers [81]; Booth-Kooley et al. proposed that this occurs because enlisted personnel reenter society without close connections to military members with similar experiences, or because military deployment has a greater negative impact on home life for reservists than for officers. Compared to Army personnel, those in the Marines, Air Force, Navy, or Coast Guard are significantly less likely to report symptoms of PTSD [61]. Few studies have examined military occupation in relation to combat exposure, or how occupation mediates links between combat exposure and mental health. In one of the few studies conducted in this area, Mayo et al. indicated that combat specialists were at a greater risk for new-onset PTSD compared with health specialists, service supply, and functional personnel [39]. It was speculated that these difference were found because military persons have more and longer deployment. Thus, military persons were at a higher risk for an enemy attack and increased combat exposure; this put them at higher risk for the development of PTSD.

Although smoking status was not positively related to PTSD in this meta-analysis, Barrett indicated that current and former smokers were more likely to meet the criteria for PTSD when compared with those who reported never smoking [61]. The association between smoking and PTSD has been previously found among U.S. veterans of the Vietnam War [2]. However, most studies focusing on smoking and other adverse health behaviors, such as alcohol and drug use, use self-report screeners rather than more structured diagnostic approaches; therefore, there could be a possible bi-directional effect. That is, those who smoke may have more PTSD symptoms or PTSD symptoms may lead them to smoke. This factor remains unclear and merits further study.

Pre-trauma factors (i.e., life events, pre-trauma psychological problems, and prior exposure to trauma) were also related to the development of combat-related PTSD. However, these findings were somewhat different from those reported in the meta-analysis by Brewin (2000). Specifically, Brewin (2000) found that the effects of pre-trauma factors were mediated by subsequent trauma factors or individual responses to the trauma. That is, the effects of these variables are distal rather than proximal, and their impact is diluted by one or more intervening variables [3].

There is a common thread between prior exposure to adverse life events and the exposed person’s own psychological problems as predictors of PTSD. Psychological problems—which can both create and result from poorer social support—play some role in the risk of PTSD after exposure to traumatic stressors [26]. Most studies have used cross-sectional or retrospective data. These studies do not have pre-deployment data on the mental health status of military samples, and only a small number of studies have examined the relationship between existing disorders and combat-related PTSD. Thus, it is difficult to ascertain the effects of decreased mental or physical health on the onset or persistence of symptoms of PTSD. Some studies have also found that prior sexual abuse may increase military person’s vulnerability or resilience following potentially traumatic events [26]; since women are more often exposed to sexual abuse than men, this finding may also help to explain the heightened PTSD prevalence among women.

### Peri-trauma factors

Deployment-related variables like combat exposure were significantly associated with combat-related PTSD. Deployed personnel with combat exposure reported more symptoms of PTSD than those deployed individuals without combat exposure. This is similar to previous findings that PTSD risk increased among combat soldiers who returned from Vietnam, the Persian Gulf, Iraq and Afghanistan, who had increased combat exposure, multiple deployments, and longer deployments [5, 82, 83]. Researchers have also reported that greater elapsed time from military active duty worry about family life during deployment, and separation from military service were significantly risk factors for PTSD [50, 84].

Although combat exposure is considered the leading stressor of war, many researchers have emphasized the importance of deployment-related stressors on PTSD; these deployment-related stressors include excessive heat or cold, concerns or problems with family members back home, boredom, lack of privacy, and problems with leadership [6]. In addition, Engekhard et al. indicated that noncombat stressors such as operational stressors, low-magnitude stressors, and contextual stressors also play an important role in the development of PTSD. However, service component difference did not emerge in this meta-analysis.

Military personnel were more likely to develop PTSD if they discharged a weapon or witnessed an injury or death during their deployment. Discharging a weapon is likely to generate a traumatic memory directly associated with the negative event, this is considered an important precursor to combat-related PTSD. It is likely that military service members who witnessed someone being injured or killed during deployment experienced intense fear at that time.

In addition, the relationship between being injured and PTSD may be related to the severity of the injuries (e.g., injuries resulting in amputation and disability). The onset of disability is likely to reduce the quality of life in military personnel and veterans that may lead to PTSD. This is consistent with what Trickey et al.’s findings; specifically, they reported that trauma severity was strongly associated with the risk of developing PTSD. However, they also indicated that the effect of trauma severity was complex, since the objective measurement was complicated by a number of conceptual factors [11]. Other studies have also demonstrated a link between physical injury and PTSD. For example, in a recent meta-analysis, Ozer et al. indicated that the strongest predictors of PTSD were psychological processes during the traumatic event [26]. MacGregor confirmed that injuries occurring in battle were more strongly associated with PTSD when compared with non-battle injuries [38].

### Post-trauma factors

Post-deployment support, subsequent life stress, and comorbid psychological problems are among the personal factors that have been considered as possible risk factors for PTSD [85]. A positive recovery environment after trauma exposure may serve as a protective factor. For instance, social support is associated with a lower PTSD risk in both the general population and military settings [3, 26]. High levels of social care and support may foster feelings of self-reliance and self-security among military people; these feelings protect against PTSD. Furthermore, military people who were unemployed after their military service were more likely to exhibit post-combat PTSD symptoms. This suggests that a loss of resources diminishes veterans’ ability to care for their families to the same extent as before the deployment. In other words, it also highlights the importance of reducing the risk of PTSD.

In the current meta-analysis, comorbid psychological problems were not risk factors for developing combat-related PTSD; thus, the relationship between comorbidities and PTSD is still unclear. In a systematic review, mixed results suggesting that comorbid psychological problems may be risk factors for PTSD following combat exposure, or that PTSD may be a predictor of comorbid psychological problems, or that they may share common risk factors [86]. However, another study by Trickey et al. revealed that comorbid psychological problems (e.g., anxiety, depression) were significant risk factors, and depression was the most predictive of PTSD [11]. Thus, future research should examine psychiatric reactions after the combat exposure.

### Limitations

It is important to note that our study had some limitations. To begin, this meta-analysis only included observational studies; most data were based on self-report measures, that can be prone to biases in sample selection, recall, and information evaluation, as well as confounding biases. There was also significant heterogeneity among the studies due to sampling, design, measurement, and statistical analysis. This is not uncommon in reviews of observational studies [87]. Furthermore, many of the variables included in the analyses were only examined in a small proportion of studies, thereby limiting the generalizability of the findings. Moreover, the 32 studies we included contained data from only a small number of female participants who were deployed to combat areas, as previously noted, women may have unique risk factors for PTSD that were not confirmed in our article, including experiences of sexual assault and childhood sexual abuse. This should be examined more closely in future research.

In addition, all of the studies involved deployed personnel and assumed that trauma exposure was combat related. However, the nature of the traumas leading to PTSD cannot be confirmed, given the self-report nature of the studies. Finally, we were not able to reveal the exact nature of the relationship between the risk factors; therefore, better levels of prediction could occur by aggregating these risk factors. Thus, investigations of interaction, mediator, and moderator effects are necessary. Nevertheless, our study highlights areas that will benefit from further investigation.

## Conclusion

In conclusion, despite some limitations of the studies included in the meta-analysis, this study revealed positive relationships between several predictors and PTSD among military personnel and veterans. As previously noted, beyond the traumatic event itself, individual and social vulnerability factors influence the development of PTSD. In our study, we examined the risk factors for PTSD based on the ORs/RRs and 95% CIs reported in each study; our analysis revealed that the relatively larger estimated effect size of predictors were adverse peri-deployment events (e.g., discharged a weapon, saw someone wounded/killed), trauma severity, and adverse life events. Thus, more attention should be given to the role of personal and environment variables in predicting PTSD.

In general, the present study has implications for the theoretical understanding of PTSD and can help military health service workers reduce the incidence of PTSD among military service members. First, this research may provide ideas for military leaders and policy makers to establish programs to improve assessments, prevention efforts, and interventions for combat-related PTSD. Specifically, it is necessary to understand the health of military personnel since assessing the mental health of military personnel before their deployment is easy to initiate. Indeed, military leaders and society should provide continuous support to military persons before, during, and after their deployment. Second, these results can help clinicians understand the relevant risk factors and provide treatment prior to difficulties becoming chronic. Additionally, clinicians should pay more attention to previously existing mental disorders in military personnel.

Importantly, there are some issues that require further study. First, the varying effects of ages, ethnicity, and branch of service should be explored in future research. Second, and the most importantly, the thrust of future research should be on the more proximal mechanisms or processes in the development of combat-related PTSD; moreover, researchers should work to better understand how these variables interact and how to better protect military personnel from combat-related PTSD.

## Supporting Information

S1 PRISMA Checklist (PDF)
